# Mining of a phospholipase D and its application in enzymatic preparation of phosphatidylserine

**DOI:** 10.1080/21655979.2017.1308992

**Published:** 2017-05-16

**Authors:** Wen-Bin Zhou, Jin-Song Gong, Hai-Juan Hou, Heng Li, Zhen-Ming Lu, Hong-Yu Xu, Zheng-Hong Xu, Jin-Song Shi

**Affiliations:** a School of Pharmaceutical Science, Jiangnan University, Wuxi, PR China; b National Engineering Laboratory for Cereal Fermentation Technology, Jiangnan University, Wuxi, PR China

**Keywords:** Cloning and expression, Enzyme characterization, Genome mining, Phosphatidylserine, Phospholipase D

## Abstract

Phosphatidylserine (PS) is useful as the additive in industries for memory improvement, mood enhancement and drug delivery. Conventionally, PS was extracted from soybeans, vegetable oils, egg yolk, and biomass; however, their low availability and high extraction cost were limiting factors. Phospholipase D (PLD) is a promising tool for enzymatic synthesis of PS due to its transphosphatidylation activity. In this contribution, a new and uncharacterized PLD was first obtained from GenBank database via genome mining strategy. The open reading frame consisted of 1614 bp and potentially encoded a protein of 538-amino-acid with a theoretical molecular mass of 60 kDa. The gene was successfully cloned and expressed in *Escherichia coli*. Its enzymatic properties were experimentally characterized. The temperature and pH optima of PLD were determined to be 60°C and 7.5, respectively. Its hydrolytic activity was improved by addition of Ca^2+^ at 5 mM as compared with the control. The enzyme displayed suitable transphosphatidylation activity and PS could be synthesized with L-serine and soybean lecithin as substrates under the catalysis of PLD. This PLD enzyme might be a potential candidate for industrial applications in PS production. To the best of our knowledge, this is the first report on genome mining of PLDs from GenBank database.

## Introduction

Phosphatidylserine (PS) is a natural phospholipid that plays an important role in the food, cosmetic and pharmaceutical industries.^1^ It is used as the component of liposome in drug delivery system, as the surfactant for cosmetics, and as the dietary supplement for foods.^2^ Furthermore, PS could be able to improve memory performance in the brain functions of elderly patients with Alzheimer disease,^3^ Parkinson disease,^4^ dementia,^5^ epilepsy,^6^ and geriatric depression.^7^ PS also has some benefits on mood enhancement from stress due to structural and functional changes in the lipid composition of the neuronal membranes in the human brain.^8-10^ Additionally, PS has been used to prevent muscle soreness, and reduce circulating cortisol concentrations during and after exercise, including cycling, weight training and endurance running.^11^ The possible sources for obtaining PS are animal organs such as a bovine brain,^12^ but these sources might not be suitable for human use due to the risk of transmission of infectious diseases. Natural PS can also be prepared from soybeans, vegetable oils, egg yolk, and biomass; however, their low availability and high extraction cost limited the industrial-scale production of PS. In literature, enzymatic conversion of phosphatidylcholine (PC) via phospholipase D (PLD) was proven potential in PS preparation.^13,14^ Notably, PC is abundant in soybean lecithin, which is widespread and relatively inexpensive. Thus, the bioconversion of soybean lecithin via PLD might be an favorable method for PS preparation.

Phospholipase D (PLD), which is an extracellular enzyme belonging to the PLD superfamily.^15^ At present, the study of PLD is mainly focused on its physiologic functions such as lipid metabolism, signal transduction and biofilm formation. PLD can also be used for the synthesis of PS according to the literature,^16^ but the relevant reports are still relatively small. PLD catalyzes 2-step reactions: (i) the hydrolysis of phosphatidylcholine to phosphatidic acid (PA) and choline via the cleavage of its phosphodiester bond and (ii) a transfer reaction in which the phosphatidic acid moiety is transferred to an acceptor alcohol (transphosphatidylation).^6,7^ PLD is widely distributed in animals, plants, and microorganisms.^17^ Even though cabbage PLD is the most abundant in nature, yet it shows a very low transphosphatidylation activity.^18^ In contrast, microbial PLDs, especially those from strains of *Streptomyces* genus, turned out to exhibit a generally higher transphosphatidylation activity, compared with the enzymes from other organisms.^19,20^ Recently, PLDs from *Streptomyces* species have been investigated for their possible application in PS production.^16^ Several approaches have been developed to discover potential PLDs. Among these approaches, the conventional screening and metagenome strategies have been widely used. However, these methods require screening a large number of strains or clones, and are thereby time-consuming. In addition, the target of these methods is not clear, and the activity of obtained PLD is relatively low. Considering that the number of genes increased exponentially based on genome sequencing and annotation in the database, genome mining has drawn considerable attention in recent years. Researchers can easily obtain genes harboring desired properties by retrieving the defined function from various public databases, such as GenBank, Pfam, and Brenda. Similarly, PLDs with potentially high phosphatidylation activity can be efficiently explored via genome mining and functional analysis.

In this study, genome mining was introduced for discovery of a potential PLD to catalyze the synthesis of PS. A novel *PLD* gene was mined from GenBank database. It was cloned and heterologously expressed in *Escherichia coli* BL21(DE3) and its catalytic properties was investigated in detail. Furthermore, PS synthesis was attempted using soybean lecithin and L-serine as substrates under the catalysis of PLD. And this is the first report on genome mining of PLDs from GenBank database.

## Results and discussion

### Discovery of target genes via genome mining

**Figure 1.**
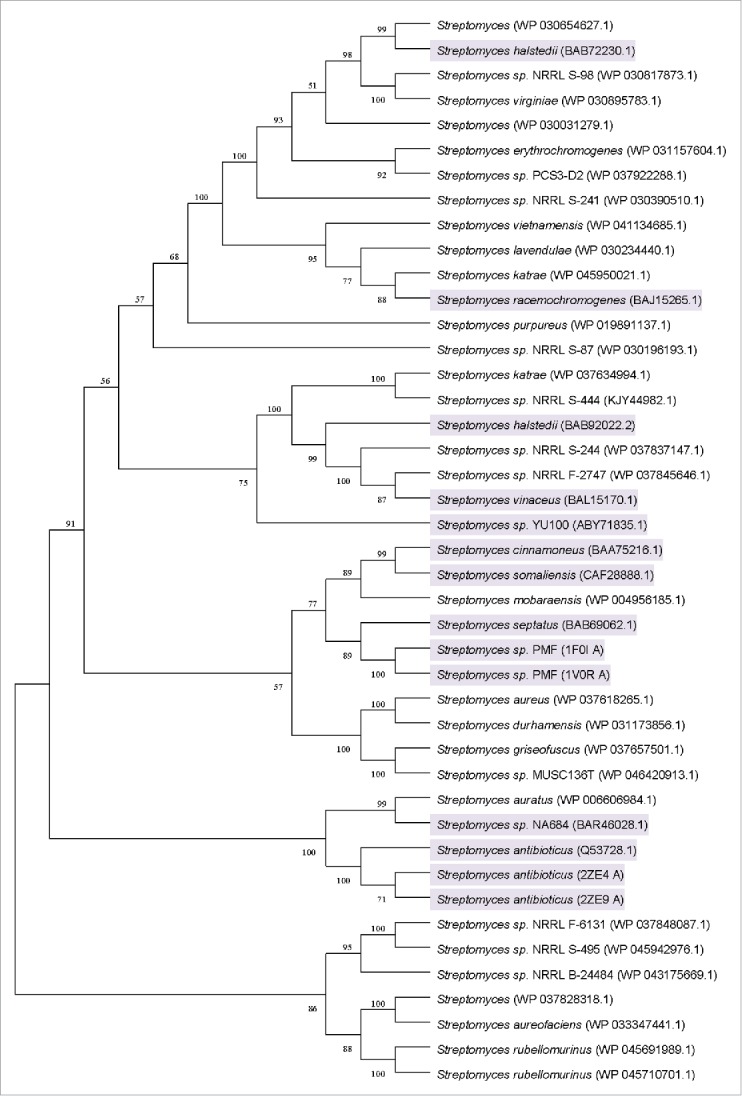
Unrooted neighbor-joining tree based on the amino acid sequences of the organisms from Table 1 (accession numbers are in parentheses). A consensus tree was constructed using a bootstrap test with 1000 replications. Bootstrap values greater than 50% are shown at the branch points. The organisms harboring defined PLD activity are shadowed.

Based on the screening criteria mentioned in genome mining and sequence analysis section, a total of 42 proteins were chosen for the transphosphatidylation activity assay (Table 1). Among the 42 proteins, 14 have been experimentally determined to harbor phospholipase D activity. Finally, a cluster containing 6 proteins was found based on phylogenetic analysis (Fig. 1). The phospholipase D from *Streptomyces* sp*.* PMF^21^ was chosen as the identifier because of its high transphosphatidylation activity, and the phospholipase D from *Streptomyces septatus*,^14^ *Streptomyces somaliensis*,^22^ *Streptomyces cinnamoneus*^13^ have also been reported to display transphosphatidylation activity. Only one protein was not characterized experimentally within this cluster, and its functions remained unclear. Based on the defined phospholipase D, this cluster was designated as the predicted phospholipase D subgroup. The sequence analysis showed that the uncharacterized PLD, which belongs to *Streptomyces mobaraensis*, showed 71.51% of identity with that from *Streptomyces* sp PMF^21^; moreover, it exhibited 76.57%, 78.19% and 79.70% of identities with the PLDs from *Streptomyces septatus*,^14^ *Streptomyces somaliensis*^22^ and *Streptomyces cinnamoneus*,^13^ respectively. The target fragment contained an open reading frame of 1614 bp in length which encoded a protein of 538-amino-acid. Its theoretical molecular weight was 60 kDa predicted by Protparam program. To the best of our knowledge, the present study is the first report on genome mining of PLDs for PS synthesis.

**Table 1.** Organisms and accession numbers of the putative phospholipase D minedfrom the GenBank database.

Organisms	GenBank Accession no.	Predicted function	Defined^b^ function	Identity^a^ (%)
*Streptomyces* sp PMF	1F0I_A		phospholipase D	100
*Streptomyces septatus*	BAB69062.1		phospholipase D	86
*Streptomyces vinaceus*	BAL15170.1		phospholipase D	83
*Streptomyces somaliensis*	CAF28888.1		phospholipase D	82
*Streptomyces cinnamoneus*	BAA75216.1		phospholipase D	82
*Streptomyces* sp NRRL S-87	WP_030196193.1	phospholipase		80
*Streptomyces griseofuscus*	WP_051850263.1	phospholipase		79
*Streptomyces* sp NRRL S-244	WP_051696556.1	phospholipase		79
*Streptomyces* sp YU100	ABY71835.1		phospholipase D	79
*Streptomyces* sp NRRL F-2747	WP_037845646.1	phospholipase		79
*Streptomyces halstedii*	BAB92022.2		phospholipase D	79
*Streptomyces lavendulae*	WP_030234440.1	phospholipase		78
*Streptomyces* sp MUSC136T	WP_046420913.1	phospholipase		78
*Streptomyces mobaraensis*	WP_004956185.1	phospholipase		77
*Streptomyces purpureus*	WP_019891137.1	phospholipase		77
Streptomyces sp NRRL S-444	KJY44982.1	phospholipase		76
*Streptomyces katrae*	WP_037634994.1	phospholipase		76
*Streptomyces racemochromogenes*	BAJ15265.1		phospholipase D	76
*Streptomyces vietnamensis*	WP_052499544.1	phospholipase		76
*Streptomyces*	WP_030031279.1	phospholipase		75
*Streptomyces katrae*	WP_045950021.1	phospholipase		75
*Streptomyces erythrochromogenes*	WP_031157604.1	phospholipase		75
*Streptomyces*	WP_030654627.1	phospholipase		75
*Streptomyces durhamensis*	WP_031173856.1	phospholipase		75
*Streptomyces halstedii*	BAB72230.1		phospholipase D	74
*Streptomyces* sp PCS3-D2	WP_037922288.1	phospholipase		74
*Streptomyces virginiae*	WP_033226935.1	phospholipase		74
*Streptomyces* sp NRRL S-241	WP_030390510.1	phospholipase		74
*Streptomyces* sp NRRL S-98	WP_030817873.1	phospholipase		74
*Streptomyces auratus*	WP_006606984.1	phospholipase		73
*Streptomyces* sp NA684	BAR46028.1		phospholipase D	73
*Streptomyces aureus*	WP_037618265.1	phospholipase		73
*Streptomyces antibioticus*	Q53728.1		phospholipase D	73
*Streptomyces antibioticus*	2ZE4_A		phospholipase D	73
*Streptomyces antibioticus*	2ZE9_A	phospholipase		73
*Streptomyces rubellomurinus*	WP_052707075.1	phospholipase		71
*Streptomyces rubellomurinus*	WP_045710701.1	phospholipase		71
*Streptomyces* sp NRRL S-495	WP_045942976.1	phospholipase		71
*Streptomyces* sp NRRL F-6131	WP_037848087.1	phospholipase		69
*Streptomyces aureofaciens*	WP_033347441.1	phospholipase		68
*Streptomyces* sp NRRL B-24484	WP_052391292.1	phospholipase		68
*Streptomyces*	WP_037828318.1	phospholipase		68

^a^BLASTP was performed by comparing the amino acid sequence with that of the phospholipase D from Streptomyces sp. PMF.

^b^The enzymes were experimentally characterized to harbor phospholipase D activity.

### Heterologous expression and Western blot analysis

**Figure 2.**
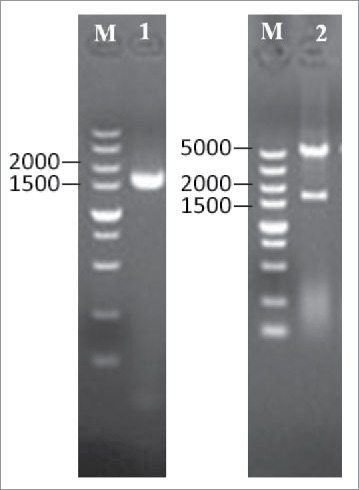
Subcloning of *PLD* gene into pET-28a(+) vector. The *PLD* gene was amplified by PCR using the primers containing restriction sites of *Bam*HI and *Hind*III and subcloned into pET-28a(+) expression vector. Lanes:M, 5 kb DNA ladder; 1, amplified *PLD* gene; 2, pET28a(+)-*PLD*/(*Bam*HI + *Hind*III).

The selected PLD encoding gene was amplified for heterologous expression in *E. coli.* A forward primer with *Bam*HI site and a reverse primer with *Hind*III site were used for gene amplification, and the amplified gene (1.6 kb) was inserted into pET-28a(+) vector. The plasmid DNA from positive transformants was confirmed by restriction digestion (Fig. 2).

**Figure 3.**
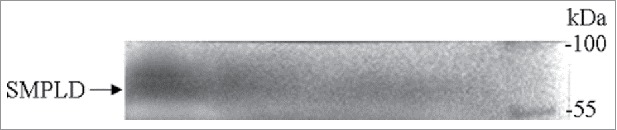
Western blot analysis for confirming the expression of PLD.

The positive clone of pET-28a(+)-PLD plasmid was cultured in LB broth supplemented with 50 μg/mL kanamycin, incubated at 37°C until the OD_600_ reached 1.0 and cultured for another 12 h at 25°C with addition of 0.5 mM IPTG. The PLD recombinant protein was well expressed in *E. coli* with a molecular mass of approximately 60 kDa through Western blot analysis, which was in agreement with the predicted value of encoding gene (Fig. 3). It was observed that the recombinant strain harboring PLD exhibited a hydrolytic activity of 0.5 U/mL, while no hydrolytic activity was detected in another control experiment which expressed the plasmid pET-28a(+) in *E. coli*. In literature, the recombinant *S. antibioticus* PLD expressed in *E. coli* XL1-Blue was determined to harbor an activity of 0.2 U/mL under the induction of IPTG.^23^ For the reasons of the low expression level of enzyme in *E. coli*, it is speculated that the PLD is toxic to host cells, resulting in cell damage immediately upon expression of active PLD, which likely degrades phospholipids contained in the membrane of the host strain.^24^

### Effects of pH on activity and stability of PLD

**Figure 4.**
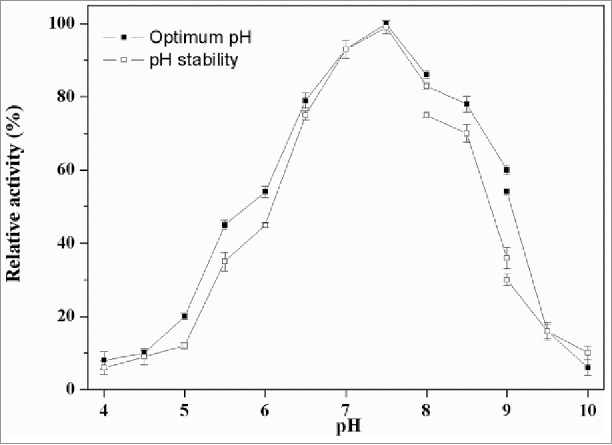
Effect of pH on PLD activity. The optimal activity of PLD was determined using standard assay in 40 mM of the following buffers. Symbols: diamond, disodium hydrogen phosphate-citric acid buffer (pH 4.0–8.0); square, Tris-HCl buffer (pH 8.0–9.0); triangle, glycine-sodium hydroxide (pH 9.0–10.0). The maximum activity was taken as 100%. To determine pH stability, the enzyme was incubated at 37°C for 60 min in various buffers mentioned above, and the residual activities were measured.

The optimum pH for PLD-catalyzed hydrolysis of PC was determined to be 7.5. As shown in Figure 4, the PLD activity gradually increased from pH 4.0 to 7.5 and decreased rapidly from pH 7.5 to 10.0, while only low activity could be detected below pH 4.0 and above 10.0. This PLD showed suitable hydrolyzing activity under neutral conditions compared with acidic or basic conditions, which was similar to other reported microbial PLDs from *Streptomyces* PMF and *Streptomyces* sp CS-57.^21,25^ The pH stability profile indicated that the PLD was stable in the pH range between 6.5 and 8.5, but underwent obvious decrease in activity when pH value was increased up to above 9.0. Furthermore, the enzyme exhibited different relative activities under the same pH condition, which may be attributed to the diverse effects of ions in different buffer solutions.

### Effects of temperature on activity and stability of PLD

**Figure 5.**
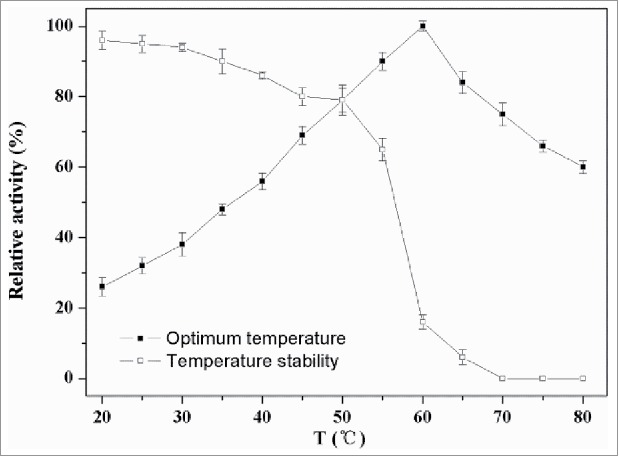
Effect of temperature on activity of PLD. The optimal activities of PLD were tested at different temperatures (20–80°C) in Tris-HCl (40 mM, pH 7.5) using standard assay. For evaluating the thermostability, the residual activities were measured in 40 mM Tris-HCl (pH 7.5) at 37°C after the enzyme was treated for 60 min at different temperatures.

The highest activity of the recombinant PLD was found at the temperature of 60°C. As shown in Figure 5, the activity of PLD kept increasing until 60°C, and softly decreased at higher temperatures. This PLD was stable up to 55°C but rapidly inactivated above 60°C. The residual enzymatic activity at 50°C and 55°C after 60 min was 79% and 65%, respectively. Generally, the reaction rates increase as the reaction temperature rises because the risen temperature increases the molecular free energy which makes more efficient collisions between the molecules. However, excessively higher temperature would influence the protein structure, which results in the reduction of enzyme activity. It was reported that PLD from *Streptomyces* PMF was stable between 20°C and 50°C; however, the residual activity obviously reduced above 50°C and it was 50% at 55°C.^21^ The residual activity of PLD from *Streptomyces halstedii* K1 remained above 60% between 15°C and 45°C but rapidly decreased at 55°C.^14^

### Effects of inhibitors and metal ions on PLD activity

The inhibitory effects of the various compounds on enzyme activity are listed in Table 2. In this study, 2-ME and DTT slightly inhibited the PLD by 25% and 20%, respectively, while its activity was obviously inhibited by SDS and PMSF, both of which reduced the activity by over 50%. It was almost completely inhibited by EDTA, the well-known metallo enzyme inhibitor, which might suggest that metallic cofactors were required for its function of PLD.

**Table 2.** Effects of various inhibitors and metal ions on PLD stability.

Inhibitors/metal ions	Concentration (mM)	Residual activity (%)	Inhibitors/metal ions	Concentration (mM)	Residual activity (%)
None	0	100 ± 0.3	Co^2+^	5	117.8 ± 1.8
2-ME	5	75 ± 0.9	Na^+^	5	101.5 ± 1.2
DTT	5	80 ± 1.1	Cu^2+^	5	108.2 ± 0.6
SDS	5	50 ± 2.1	Mn^2+^	5	101.7 ± 1.5
PMSF	5	40 ± 0.3	Li^+^	5	107.6 ± 0.8
EDTA	5	6 ± 0.6	Fe^3+^	5	105.9 ± 2.1
K^+^	5	105.2 ± 1.5	Fe^2+^	5	56.0 ± 2.2
Mg^2+^	5	101.1 ± 0.3	Ag^+^	5	27.0 ± 1.2
Ba^2+^	5	118.2 ± 1.5	Zn^2+^	5	109.7 ± 0.6
Ca^2+^	5	134.1 ± 0.2	Al^3+^	5	109.3 ± 2.4

*Note.* The activity of control sample without addition of any chemicals was considered as 100%. Residual activity was measured at pH 7.5 at 55°C.

Several metal ions were also assayed for their effects on PLD activity (Table 2). Na^+^, Mn^2+^ and Mg^2+^ showed no significant influence on the enzyme activity. The addition of Ba^2+^ and Co^2+^ at 5 mM improved the activity to 118% and 117%, respectively. Especially, Ca^2+^ displayed the most dramatic effect on enzyme activity, which was increased by 34%. In literature, Ca^2+^ also displayed favorable effect on catalytic synthesis of PS.^25,26^ Moreover, in several reports, PLD activity assays were performed at different concentrations of calcium ions in the reaction mixtures as observed for PLD from *Streptomyces* sp P821 with 15 mM CaCl_2_^16^, PLD from *Streptomyces* sp with 50 mM CaCl_2_,^27^ and PLD from *Streptomyces* sp YU100 with 1.0 M CaCl_2_.^28^ Both Ca^2+^-dependent and Ca^2+^-independent PLD enzymes have been found in mammals,^29^ yeasts,^30^ and actinomyces,^31,32^ whereas almost all plant PLDs required Ca^2+^ for stimulation of the PLD activity. Moreover, the enzyme was slightly activated by K^+^, Cu^2+^, Li^+^, Fe^3+^, Al^3+^ and Zn^2+^ and severely inhibited by Fe^2+^ and Ag^+^. However, K^+^ and Zn^2+^ showed detrimental effect in previous articles.^25^

### Enzymatic preparation of phosphatidylserine (PS)

**Figure 6.**
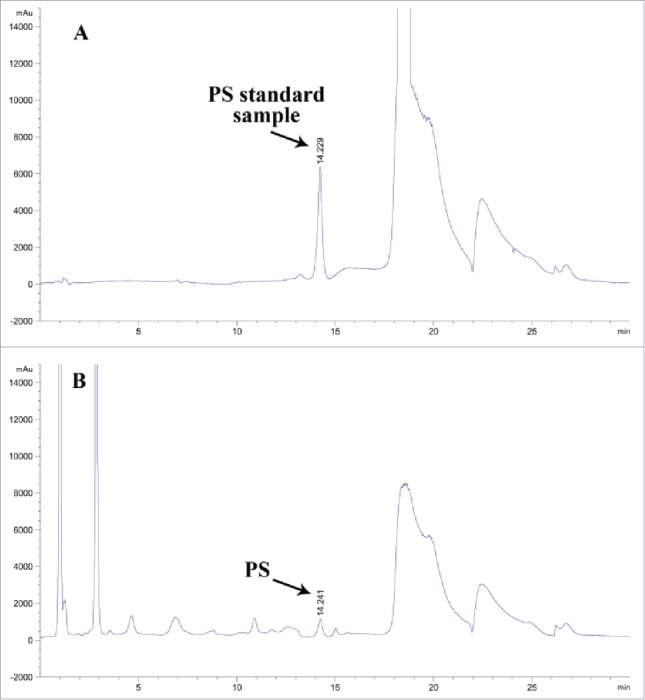
Determination of PS by HPLC. The reaction mixtures were subjected to HPLC using ELSD detection for the determination of PS. (A) The PS standard sample. (B) The reaction mixture for 10-h transphosphatidylation.

The catalytic performance of PLD was attempted for synthesis of PS with L-serine and soybean lecithin as the substrates. The reaction mixture consisting of soybean lecithin (10 mg/mL) dissolved in 8 mL of organic solvent (ethyl acetate), 30 mg/mL L-serine and 15 mM CaCl_2_ with 1.2 U PLD resuspended in 40 mM Tris-HCl (pH 7.5) was incubated at 40°C, 180 rpm for 10 h. PS was determined using Shimatzu HPLC 10 VP series equipped with ELSD detection. As shown in Figure 6, 0.2 g/L PS was observed in the transformation mixture at 14.2 min, while no PS was detected in the control sample. The Internal Standard Method of HPLC confirmed the generation of PS in the reaction mixture. The results also suggested that PLD harbored favorable transphosphatidylation activity. Further culture optimization are undergoing in our laboratory.

## Experimental

### Materials

*E. coli* BL21(DE3) and pET-28a(+) (Novagen, China) were applied for gene expression. The recombinant plasmid pET28a(+)-*PLD* was constructed and preserved in our laboratory. *E. coli* BL21(DE3) were cultured in a Luria-Bertani medium comprising (g/L): tryptone 10, yeast extract 5 and NaCl 10, pH 7.2. Soybean lecithin(Sigma, China) and L- serine(Aladdin, China) were used as substrates for the synthesis of PS. Phosphatidylserine(Sigma, China) were purchased to be the standard. All other chemicals and reagents used were of analytical grade.

### Genome mining and sequence analysis

The database searches of sequence data were performed using the BLASTP program in NCBI. The PLD from *Streptomyces* sp PMF^21^ was chosen as the identifier to search target sequences. *Streptomyces* sp PMF enzyme has the highly conserved HxKxxxxD (HKD) motifs and high transphosphatidylation activity. The identity of the amino acid sequence was used as the first criterion to screen the BLASTP result. Several PLDs showing 30–90% amino acid sequences identities with phospholipase D from *Streptomyces* sp PMF. The second criterion was the conserved HxKxxxxD (HKD) motifs.^33^ Sequences with 2 conserved HxKxxxxD (HKD) motifs were chosen once because these sequences usually displayed transphosphatidylation activity. The third criterion was the availability of the organisms that harbor phospholipase D. Regardless of the 3 criteria, the sequences with experimentally defined phospholipase substrate specificity were chosen as a priority to refine the phylogenetic analysis. The alignment of the obtained sequences was conducted using Clustal X version 1.81.^34^ A bootstrap consensus tree was built using neighbor-joining method packaged in MEGA version 5.0.^35^

### Cloning and expression of phospholipase D gene in *E. coli*

The target gene was synthesized chemically by Sangon Biotechnology (Shanghai, China). Based on the PLD gene sequence from *S. mobaraensis* (GeneBank accession no. AORZ01000236.1), the target DNA was amplified using a polymerase chain reaction (PCR; C1000, BIO-RAD, USA) procedure with 2 primers: smpld-F, 5′-CGG GAT CCC CGA AGG ACG CAG CCT TG-3′, and smpld-R, 5′-CCC AAG CTT TTA CAG ACT ACA CAC ACC-3′ that contained *Bam*HI and *Hind*III restriction sites, respectively. The PCR conditions were as follows: denaturation at 95°C for 3 min, followed by 34 cycles of 30 s at 95°C for denaturation, 2 min at 66.6°C for annealing, and 1 min at 72°C for extension. Amplified PCR products (1.6 kb) were digested with *Bam*HI and *Hind*III, and subcloned into the pET-28a(+) vector. The ligation product was transformed into *E. coli* BL21 (DE3), and the transformed cells were spread onto LB agar plates containing kanamycin (50 μg/mL). A single colony of *E. coli* BL21 (DE3) cells harboring the plasmid pET-28a(+)-PLD was grown at 37°C for 12 h in 10 mL of LB medium containing 50 μg/mL kanamycin. An aliquot (1 mL) of this culture was inoculated into 50 mL of the same medium and grown at 37°C with shaking. When the OD_600_ value reached 1.0 (Mapad UV-1800, Shanghai, China), enzyme expression was induced by adding isopropyl-β-D-thiogalactopyranoside (IPTG) with a final concentration of 0.5 mM. After cultivation was continued for another 12 h at 25°C, cells were harvested by centrifugation (Himac CR22GII, HITACHI, Japan) at 8,000 × g for 10 min at 4°C and stored at −20°C (SANYO, Japan) for further use.

### Western blot analysis

The collected cell pellet was resuspended in 5 mL Tris-HCl (40 mM, pH 7.5). The suspensions were sonicated for 30 min (working 4 s and intervals 6 s) on ice using Ultrasonic processor (Scientz Biotechnology, JY92-II, Ningbo, China). The suspension was centrifuged at 12,000 × g for 20 min and the supernatant was collected. Proteins from the supernatant were separated on 10% SDS-PAGE gels (BIO-RAD, USA) and then electrophoretically transferred to a PVDF membrane (PALL). After blotting, the filters were incubated with primary and secondary antibodies. The antibodies used were: His-tag monoclonal antibody (Novagen) at 1:1000; anti-rabbit IgG, HRP-linked antibody at 1:2000.

### Hydrolytic activity assay

PLD hydrolytic activity was typically measured by spectrophotometric assay using PC as the substrate.^25^ The reaction mixture (total volume, 100 µL) consisted of 0.5% (w/v) soybean lecithin, 0.1% (v/v) Triton X-100, 40 mM Tris–HCl (pH 7.5), 10 mM CaCl_2_ and 40 µL of an enzyme sample. After incubation at 55°C for 20 min, the reaction was terminated by addition of 50 µL solution containing 50 mM EDTA and 100 mM Tris-HCl (pH 7.5), and the PLD enzyme was immediately denatured by heating at 100°C for 5 min. After cooling the reaction mixture to room temperature for 5 min, 500 µL of 50 mM Tris–HCl (pH 7.5) containing 1 mg phenol, 0.3 mg 4-aminoantipyrine, 3 U/mL of choline oxidase (Sigma, China), and 2 U/mL of horseradish peroxidase (Sigma, China) was added. After incubation at 37°C for 20 min, the absorbance of the reaction mixture was measured at 505 nm (Multiskan Ascent, ThermoFisher, USA). The calibration curve was obtained by using a standard solution of choline chloride instead of the enzyme solution. One unit (U) of hydrolytic activity of PLD was defined as the amount of enzyme that produced 1 µmol choline per min. All assays were performed in triplicate.

### Effects of pH on the activity and stability of PLD

The activity of purified PLD was measured at pH range of 4–10 at 37°C using soybean lecithin as substrate. Its pH stability was determined by preincubation in buffer solutions at 37°C and different pH values for 60 min. Aliquots were withdrawn, and residual enzymatic activity was determined at pH 7.5 and 37°C. The following buffer systems were used at 50 mM: glycine-HCl at pH 4 and 5; monopotassium phosphate-NaOH at pH 6 and 7; Tris-HCl at pH 8–9, glycine-NaOH at pH 10.

### Effect of temperature on the activity and stability of PLD

The effect of temperature on PLD activity was examined at 20–80°C and pH 7.5 for 20 min. Thermal stability was determined by incubation at different temperatures (20, 30, 40, 50, 60, 70, and 80°C) and pH 7.5 for 60 min, and then cooled in the ice immediately. Aliquots were withdrawn at regular intervals to test the remaining activity under standard conditions. The non-heated enzyme, which was cooled in ice, was considered as the control (100%).

### Effects of inhibitors and metal ions on PLD stability

The effects of 2-mercaptoethanol (2-ME), DL-dithiothreitol (DTT), sodium dodecyl sulfonate (SDS), phenylmethanesulfonyl fluoride (PMSF), ethylene-diaminetetraacetic acid (EDTA), and various monovalent and divalent metal ions (5 mM and 10 mM) on enzyme stability were investigated by pre-incubating the enzyme for 60 min at 37°C with these chemicals. Enzyme assays were performed under standard assay conditions.

### Enzymatic preparation of phosphatidylserine(PS) by crude enzyme

The reaction mixture consisting of soybean lecithin (10 mg/mL) dissolved in 8 mL of organic solvent (ethyl acetate), 30 mg/mL L-serine and 15 mM CaCl_2_ in 4 mL crude enzyme solution resuspended in 40 mM Tris-HCl (pH 7.5) was incubated at 40°C and 180 rpm for 10 h. PS was detected by HPLC (Shimatzu HPLC 10 VP series, Japan) equipped with ELSD detection. Reaction mixture was eluted with a LiChrosphere 100 diol(4µm × 125 mm, Merk, USA) using solvent A (hexane/2-propanol/acetic acid/triethylamine, 820: 170: 10: 0.8, by vol.) and solvent B (2-propanol/water/acetic acid/triethylamine, 850: 140: 10: 0.8, by vol.). The elution profile was as follows: 0 min, B = 5%; 0–23 min, B increased to 40%; 23–28 min, B increased to 100%; 28–29 min, B = 100%; 29–39 min decreased to 5%.

### Statistical analysis

All the assays were performed in triplicate in this study. The mean standard deviation (± SD) was used for the data processing, which was analyzed using GraphPad Prism 5 (San Diego, CA, USA).

## Conclusions

Based on 3 screening criteria, a new PLD was successfully mined from *S. mobaraensis* and expressed in *E. coli*, which could catalyze soybean lecithin and L-serine into PS. To the best of our knowledge, this study is the first report about PLDs from *S. mobaraensis*. The recombinant PLD displayed obvious hydrolyzing activity toward PC and its enzymatic properties was investigated. It also exhibited suitable transphosphatidylation activity and PS could be successfully synthesized under the catalysis of this PLD. These results indicate that the PLD may be potential as a biocatalyst for practical production of PS through the hydrolysis of soybean lecithin.
